# Counter-Gradient Variation in Respiratory Performance of Coral Reef Fishes at Elevated Temperatures

**DOI:** 10.1371/journal.pone.0013299

**Published:** 2010-10-11

**Authors:** Naomi M. Gardiner, Philip L. Munday, Göran E. Nilsson

**Affiliations:** 1 School of Marine and Tropical Biology, James Cook University, Townsville, Australia; 2 Australian Research Council Centre of Excellence for Coral Reef Studies, James Cook University, Townsville, Australia; 3 Physiology Programme, Department of Molecular Biosciences, University of Oslo, Oslo, Norway; Dalhousie University, Canada

## Abstract

The response of species to global warming depends on how different populations are affected by increasing temperature throughout the species' geographic range. Local adaptation to thermal gradients could cause populations in different parts of the range to respond differently. In aquatic systems, keeping pace with increased oxygen demand is the key parameter affecting species' response to higher temperatures. Therefore, respiratory performance is expected to vary between populations at different latitudes because they experience different thermal environments. We tested for geographical variation in respiratory performance of tropical marine fishes by comparing thermal effects on resting and maximum rates of oxygen uptake for six species of coral reef fish at two locations on the Great Barrier Reef (GBR), Australia. The two locations, Heron Island and Lizard Island, are separated by approximately 1200 km along a latitudinal gradient. We found strong counter-gradient variation in aerobic scope between locations in four species from two families (Pomacentridae and Apogonidae). High-latitude populations (Heron Island, southern GBR) performed significantly better than low-latitude populations (Lizard Island, northern GBR) at temperatures up to 5°C above average summer surface-water temperature. The other two species showed no difference in aerobic scope between locations. Latitudinal variation in aerobic scope was primarily driven by up to 80% higher maximum rates of oxygen uptake in the higher latitude populations. Our findings suggest that compensatory mechanisms in high-latitude populations enhance their performance at extreme temperatures, and consequently, that high-latitude populations of reef fishes will be less impacted by ocean warming than will low-latitude populations.

## Introduction

Elevated levels of atmospheric carbon dioxide are set to increase mean global temperatures by 2–4°C in the next century [1]. Ocean temperatures have already increased by 0.1°C in recent decades [2] and sea surface temperatures are predicted to increase up to 3°C within the next century [1], [3], [4]. The effect of higher temperatures on species' distribution and abundance includes range shifts, population collapses, local extinctions, and phase shifts [5]–[11]. These patterns emerge from the combined responses of populations to increasing temperature throughout the species' geographic range. Local adaptation to thermal gradients can cause populations in different parts of the geographic range to exhibit different responses to temperature variation [12]–[18]. Consequently, comparing the effects of temperature increases in different populations is essential for generating robust predictions about the impact of global warming on animal communities at large spatial scales.

**Figure 1:**
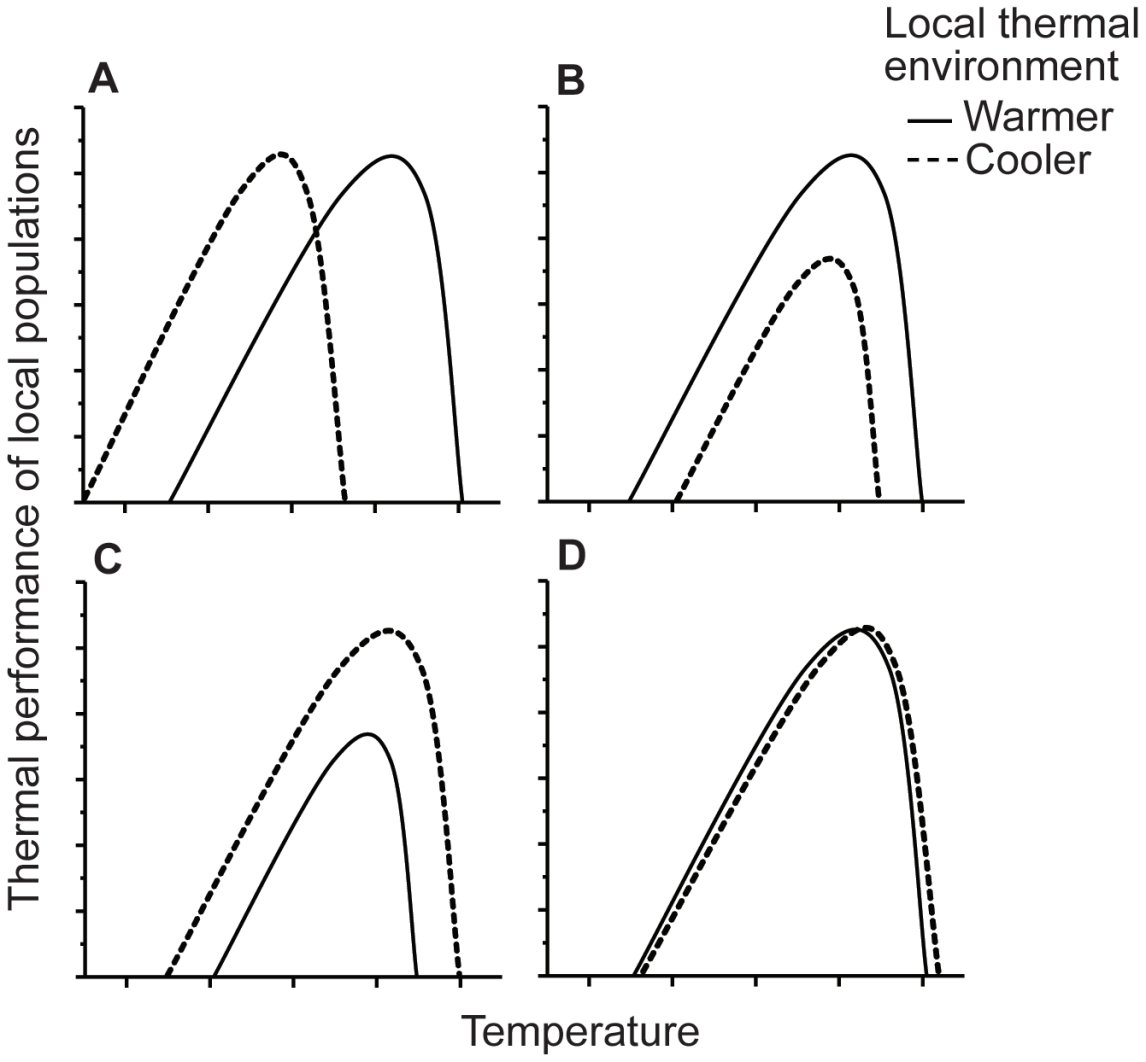
Theoretical patterns of thermal performance. A comparison of thermal performance in populations from warmer versus colder environments could exhibit (A) Local adaptation as predicted by optimality models in which thermal tolerance and optima has diverged amongst populations according to their local thermal experience. (B) Co-gradient variation whereby genetic and environmental influences are positively associated across a thermal gradient. (C) Counter-gradient variation whereby genetic and environmental influences are negatively associated across a thermal gradient or (D) No divergence in thermal performance amongst warm and cold locations. Figure modified from Fig 3.19 in Angiletta (2009) [20].

Comparing phenotypic variation in a species' performance traits across a thermal gradient could produce several patterns [19]–[22]. (1) Thermal optima could be locally adapted to match the thermal environment, such that populations from warmer locations outperform populations from cooler locations at higher temperatures, but populations from cooler locations outperform populations from warmer locations at cooler temperatures (Fig. 1a). This pattern of thermal performance would be consistent with optimality models of thermal adaptation and developmental acclimation [20]. (2) Thermal performance curves could exhibit co-gradient variation, whereby populations from warmer locations tend to outperform populations from cooler locations at all temperatures (Fig. 1b). This pattern of thermal performance is predicted when genetic and environmental influences on performance are positively associated across the thermal gradient [21]. (3) Thermal performance curves could exhibit counter-gradient variation, whereby populations from cooler locations outperform populations from warmer locations at all temperatures (Fig. 1c). This pattern of thermal performance is predicted when genetic and environmental influences on performance are negatively associated across the thermal gradient [20]. (4) Finally, there might be no difference in thermal performance curves between populations (Fig. 1d). This pattern would be predicted if populations do not acclimate to the local thermal environment and high gene flow between populations restricts local adaptation to a thermal gradient. Each of these alternatives has different implications for how species would respond to increasing average temperature across their geographic range.

In aquatic systems, comparisons of geographic variation amongst conspecific populations has predominantly focused on polar and temperate climates, and counter-gradient patterns have often been detected [22], [23]. Counter-gradient variation can arise when there is a trade-off between traits or processes in order to compensate for detrimental effects of the environmental gradient on performance, such as that induced by temperatures above a population's thermal optima [21], [22]. For example, physiological processes may be locally adapted to maintain growth and developmental rates against a thermal gradient that has a negative effect on these traits [20], [21]. Whether similar compensatory mechanisms occur in tropical marine species is largely unknown. While some tropical species appear to live close to their upper thermal limits [23], [24], the capacity to acclimate may increase species flexibility to succeed in warmer temperatures [20]. With tropical ocean temperatures predicted to increase up to 3°C over the next 100 years [3], [4], investigating the response of tropical marine taxa to higher temperatures has become increasingly important.

In water breathing animals, such as fish, a key mechanism affecting performance with increasing temperatures is aerobic capacity. The capacity of the circulatory and ventilatory systems to keep pace with higher oxygen demand at higher temperatures is thought to determine a species' viability in a warmer environment [9], [25]. Populations that can maintain their aerobic capacity at warmer temperatures have a higher thermal tolerance, and are thereby predicted to persist longer, than populations that experience a decline in aerobic performance as temperature increases. Recently Nilsson and colleagues [26] demonstrated strong inter-familial differences in thermal tolerance amongst coral reef fish species. A temperature rise of just 2°C above current summer averages caused a loss of aerobic capacity in two cardinalfish species but only slight declines in aerobic performance of three damselfish species. Their results predict rising sea temperatures will alter reef fish community structure by causing more substantial declines in cardinalfish populations than in the damselfish populations. Deleterious effects of rising temperature on aerobic performance has already led to population collapses and ecosystem shifts in polar and temperate regions [9], and similar effects might be expected to occur in tropical marine systems.

This study compares thermal performance curves of tropical fishes in two locations on the Great Barrier Reef, Australia, which are separated from each other by over 1200 km and that experience markedly different thermal regimes. Resting oxygen consumption (MO_2Rest_), maximal oxygen consumption (MO_2Max_) and aerobic scope (MO_2Max_ - MO_2Rest_) were measured for six common coral reef fishes at temperatures up to 5°C above the summer average at Heron Island on the southern Great Barrier Reef and at Lizard Island on the northern Great Barrier Reef. These locations have average summer temperatures of 27.5°C and 28.9°C, respectively, and average annual thermal ranges of 6.3°C and 4.8°C respectively. Specifically, we tested if thermal effects on respiratory performance of the six species varied between the two locations in a manner consistent with local adaptation to differences in average summer temperatures. Under this scenario, populations from the warmer, lower latitude would outperform populations from the cooler, higher latitude when exposed to warm temperatures. However, given evidence for gene flow among populations of fishes on the Great Barrier Reef [27], [28], local adaptation might be restricted and similar patterns of respiratory performance at the two locations may be expected. Alternatively, compensatory mechanisms might produce counter-gradient patterns of thermal performance. In this case populations from the cooler, high-latitude location would outperform populations from the warmer low-latitude location when exposed to warmer temperatures.

## Methods

### Ethics Statement

This research was undertaken with approval of the James Cook University animal ethics committee (permit: A1270) and according to the University's animal ethics guidelines.

### Study sites and species

Thermal effects on respiratory performance were estimated for populations of six common coral reef fishes at Lizard Island (14°40′S 145°28′E) and Heron Island reef (23°27′S, 151°57′E) on the Great Barrier Reef, Australia. Experiments were conducted during the months in which maximal summer sea surface temperatures are typically reached for each location. Northern populations were sampled between December and January in 2008 and 2009. These Lizard Island data are from Nilsson and colleagues [26] supplemented with new measurements for *Pomacentrus moluccensis*. Southern population experiments were sampled between February and March 2009 at Heron Island Research Station. Average sea surface temperatures (1982–2008) for the three warmest months (January-March) are 28.9°C in the Lizard Island region and 27.5°C in the Heron Island region (Rayner et al. 2003, 2006). The warmest months are January in the Lizard Island region (mean 29.2°C) and February in the Heron Island region (mean 27.85°C). The average range of annual sea surface temperatures (warmest monthly mean - coldest monthly mean) are 4.8°C for Lizard Island and 6.3°C for Heron Island. Temperatures were obtained from http://badc.nerc.ac.uk/data/hadisst.

Six locally abundant species of reef fish from two families were examined: two cardinalfish (*Ostorhinchus cyanosoma* and *O. doederleini*) and four damselfish (*Dascyllus aruanus*, *Chromis atripectoralis or Chromis viridis*, *Acanthochromis polyacanthus* and *Pomacentrus moluccensis*). Wet mass (g) of fish used at each location and in each temperature treatment are shown in Table 1.

**Table 1:** Mean body mass (wet weight, g) of reef fish used in metabolism experiments at each location (Heron and Lizard Island) and temperature group.

			Temperature (°C)				
Species	Location		27	29	31	32	33
*O. cyanosoma*	Heron	Mean	1.417	1.326	1.455	1.501	
		SE	0.039	0.102	0.171	0.082	
	Lizard	Mean		2.468	2.343	2.439	1.722
		SE		0.195	0.189	0.181	0.148
*O. doederleini*	Heron	Mean	2.167	2.176	1.928	2.058	
		SE	0.264	0.232	0.149	0.185	
	Lizard	Mean		1.678	2.244	2.419	1.864
		SE		0.081	0.325	0.351	0.228
*A. polyacanthus*	Heron	Mean	2.568	2.492	2.440	2.890	
		SE	0.159	0.212	0.264	0.349	
	Lizard	Mean		2.796	2.153	2.820	2.601
		SE		0.254	0.160	0.223	0.314
*C. atripectoralis*	Heron	Mean	4.136	4.561	4.520	3.242	
		SE	0.335	0.462	0.415	0.251	
	Lizard	Mean		5.165	2.402	5.641	5.113
		SE		0.318	0.337	0.337	0.332
*D. aruanus*	Heron	Mean	3.100	2.880	4.228	3.468	
		SE	0.312	0.233	0.525	0.219	
	Lizard	Mean		3.344	3.630	2.906	3.785
		SE		0.386	0.477	0.239	0.643
*P. moluccensis*	Heron	Mean	2.277	3.128	2.941	3.503	
		SE	0.401	0.307	0.305	0.494	
	Lizard	Mean		2.854	3.565	3.068	2.509
		SE		0.229	0.222	0.304	0.336

SE is standard error.

### Respirometry experiments

Fish were captured from shallow lagoon and slope areas using clove oil anesthetic solution [29], small hand nets, or a 5 m barrier net. Methods of aquarium, feeding and temperature regimes, as well as the measurement of respiratory rates followed those previously described by Östlund-Nilsson and Nilsson [30] and Nilsson et al. [26]. In brief, control and treatment fish were placed in identical 50-L aquaria and fed twice daily to satiation with commercial fish pellets. Control fish were maintained at the average summer temperatures for each location. Once fish had adjusted to aquaria conditions (commenced feeding and showed no abnormal behaviours), treatment temperatures were slowly increased over 1–3 days with aquarium heaters. Lizard Island experimental temperatures were 31, 32 and 33°C, in addition to a control temperature of 29°C. Heron Island experimental temperatures were 29, 31, and 32°C with a control temperature of 27°C. Maximal daily variation for treatment temperatures was ±0.5°C. Fish were kept at each temperature for 4–7 days before oxygen consumption was measured. A longer period was considered unnecessary since we have previously found that this has no significant effect on oxygen consumption in coral reef fish of these families [31]. Fish were starved for 24 h prior to measuring MO_2Rest_, then fed ad libitum during a 24 h rest period before MO_2Max_ was measured. It has been suggested that the aerobically fuelled muscle mass in some fish is not large enough to force them to reach the maximum rate of oxygen uptake during maximal swimming performance [32]. Therefore, fish were fed prior to estimating MO_2Max_ to ensure their oxygen demands were high enough to engage the full capacity of the respiratory system.

MO_2Rest_ and MO_2Max_ were measured for 5–12 individuals per species and location. MO_2Rest_ indicate the cost of maintaining basic metabolic functions and was measured as described by Östlund-Nilsson and Nilsson [30]. In short, one fish at a time was put in a sealed cylindrical 750-ml Perspex chamber fitted with an oxygen electrode (OXI 340i from WTW, Germany). After running water through the chamber for 1–1.5 h (or longer if the fish showed signs of agitation) it was sealed and the fall in the dissolved oxygen concentration was recorded over 45–60 min. Experiments were ceased if dissolved oxygen fell below 60%. MO_2Max_ was measured as described by Nilsson et al. [33] except that a slightly larger chamber (500 ml) was used. In short, water was set in motion in a circular chamber by a 6 cm stirrer magnet and a wire mesh separated the fish from the magnet. A central cylinder created a circular swim chamber for the fish. Water speed was increased to a point where the fish only just keep up swimming against the current using the pectoral (aerobic) swimming mode. The fall in oxygen concentration in the sealed swimming chamber was recorded for 5–10 min during which time there was a linear fall in oxygen concentration. Oxygen levels during swimming trials were between 90 and 100% of air saturation. MO_2Rest_ and MO_2Max_ were determined for each individual, but different individuals were used for each temperature and location treatment.

The effect of location on MO_2Rest_, MO_2Max_ and aerobic scope at the common treatment temperatures of 29, 31 and 32°C was compared using two-way fixed factor ANOVA. Because oxygen uptake varies with body mass, we first regressed oxygen consumption against body mass within each treatment temperature at each location. The residuals of the regressions were then used in the ANOVA. This approach accounted for any differences in body size of the test fish between locations.

The range of temperatures tested differed between locations; 27–32°C at Heron Island and 29–33°C at Lizard Island. Therefore, in order to assess the effect of temperature rise on oxygen consumption across the full temperature range used, separate one-way ANOVAs were also conducted for each location. To account for minor differences in body size among temperature treatments the residuals from a regression of oxygen consumption against body mass for each location were used in the ANOVA. Following one-way ANOVA, Dunnett's two-sided t-tests were used to compare the treatment temperatures against the control temperature (Heron = 27°C, Lizard = 29°C). Analysis of variance assumptions were verified using residual plots and Levene's test. Due to heterogeneous variances in one location for one species, a Kruskal-Wallis test was required to compare the effect of temperature on resting metabolism.

## Results

### Maximal and resting respiration

**Figure 2:**
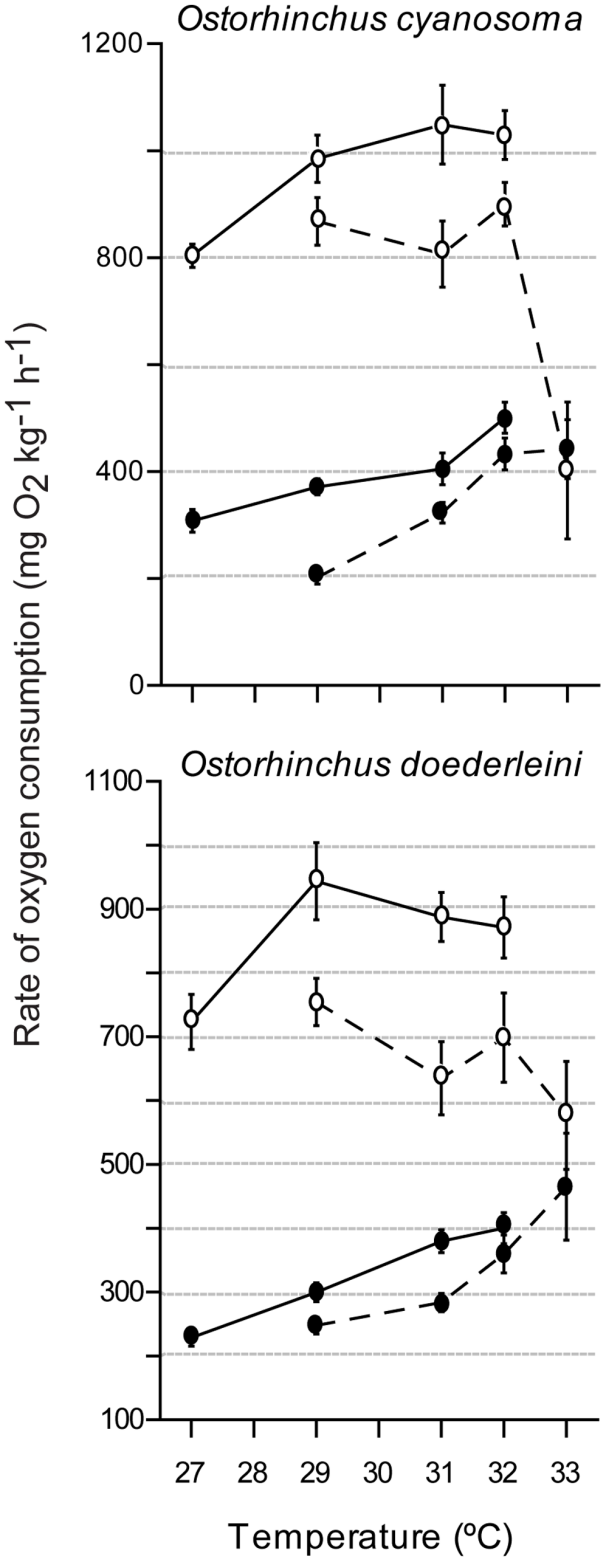
A latitudinal comparison of oxygen consumption in cardinalfish. Maximum (open circles) and resting (solid circles) rates of oxygen consumption of two cardinalfish species from a high-latitude Great Barrier Reef location (Heron Island: solid lines) and a lower latitude Great Barrier Reef location (Lizard Island: dashed lines). Values are means ± SE from 5–12 fish.

**Figure 3:**
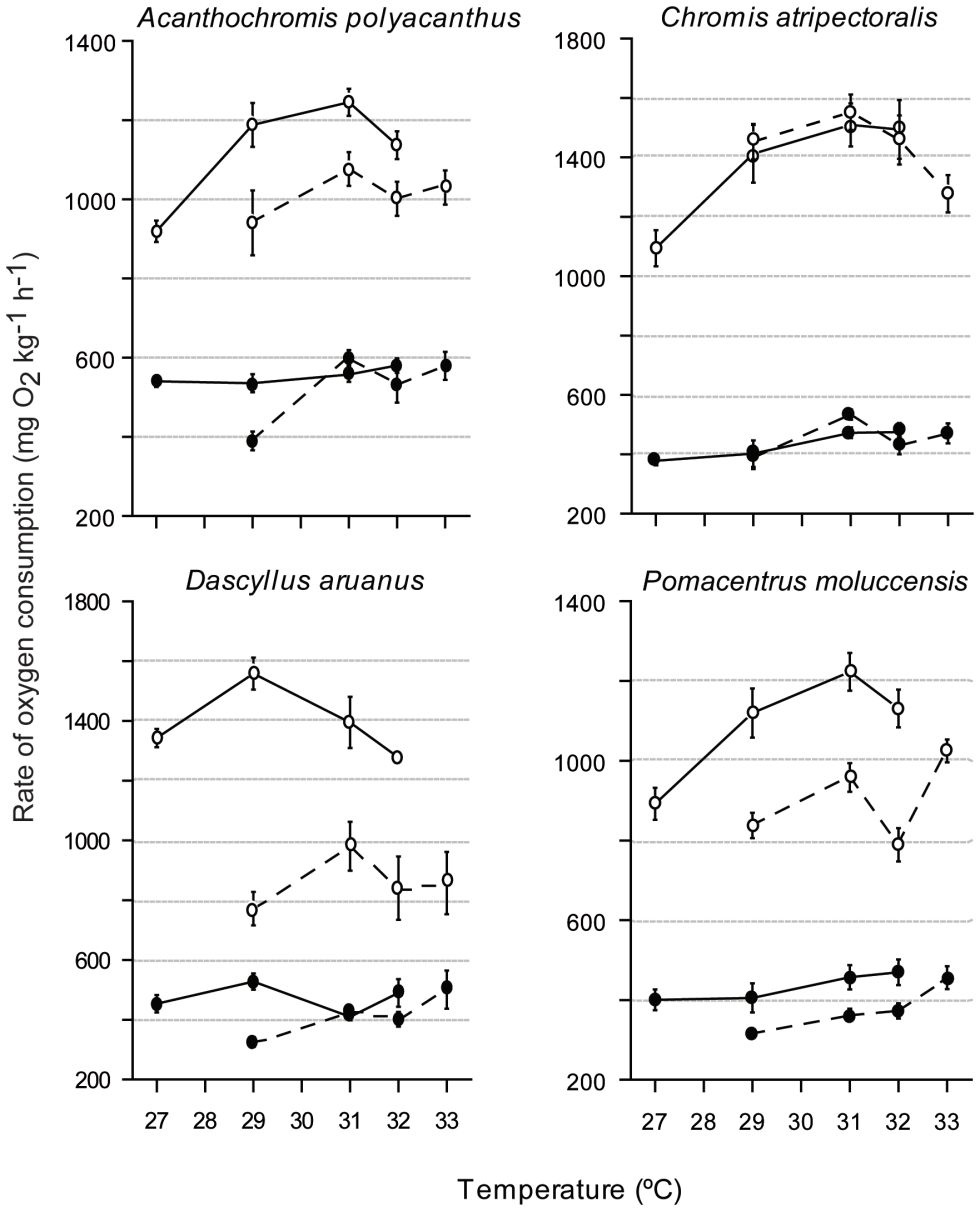
A latitudinal comparison of oxygen consumption in damselfish. Maximum (open circles) and resting (solid circles) rates of oxygen consumption of four damselfish species from a from a high-latitude Great Barrier Reef location (Heron Island: solid lines) and a lower latitude Great Barrier Reef location (Lizard Island: dashed lines). Values are means ± SE from 5–12 fish.

At common treatment temperatures, four out of the six species showed significantly higher MO_2Rest_ and MO_2Max_ at the high-latitude location (Heron Island, southern GBR) than at the low-latitude location (Lizard Island, northern GBR) (Figs. 2–3, Table 2a–b).

**Table 2:** Statistical results of two-way ANOVA analyses on the effects of latitude and increasing temperature on (a) MO_2Rest_, (b) MO_2Max_ and (c) aerobic scope for two cardinalfish species and four damselfish species.

*Species*	Source	Sum of Squares	df	Mean Square	F	p
**(a) Resting metabolism**						
**Cardinalfish**						
*O. cyanosoma*	Temperature	1416	2	708.13	0.163	0.850
	Location	2895	1	2895.39	0.667	0.420
	Temperature × Location	2165	2	1082.59	0.249	0.781
	Error	156351	36	4343.07		
*O. doederleini*	Temperature	366	2	182.91	0.093	0.911
	Location	34127	1	34126.64	17.390	**0.000**
	Temperature × Location	6833	2	3416.42	1.741	0.189
	Error	72611	37	1962.46		
**Damselfish**						
*A. polyacanthus*	Temperature	1032	2	516.25	0.140	0.870
	Location	19589	1	19588.76	5.320	**0.027**
	Temperature × Location	42420	2	21209.79	5.761	**0.007**
	Error	139909	38	3681.81		
*C. atripectoralis*	Temperature	58	2	29.22	0.005	0.995
	Location	6719	1	6719.13	1.226	0.275
	Temperature × Location	589	2	294.43	0.054	0.948
	Error	208192	38	5478.73		
*D. aruanus*	Temperature	6532	2	3265.75	0.782	0.466
	Location	53974	1	53973.61	12.931	**0.001**
	Temperature × Location	56284	2	28142.11	6.742	**0.004**
	Error	129390	31	4173.87		
*P. moluccensis*	Temperature	18	2	9.11	0.002	0.998
	Location	95162	1	95162.22	21.689	**<0.001**
	Temperature × Location	6013	2	3006.61	0.685	0.509
	Error	214992	49	4387.58		
**(b) Maximum metabolism**						
**Cardinalfish**						
*O. cyanosoma*	Temperature	6	2	3.02	0.000	1.000
	Location	4611	1	4611.21	0.242	0.626
	Temperature × Location	10493	2	5246.35	0.275	0.761
	Error	763592	40	19089.80		
*O. doederleini*	Temperature	4106	2	2052.80	0.122	0.886
	Location	400981	1	400980.79	23.772	**<0.001**
	Temperature × Location	22285	2	11142.58	0.661	0.523
	Error	624119	37	16868.07		
**Damselfish**						
*A. polyacanthus*	Temperature	1232	2	616.24	0.035	0.966
	Location	305374	1	305373.83	17.271	**<0.001**
	Temperature × Location	5363	2	2681.30	0.152	0.860
	Error	671875	38	17680.93		
*C. atripectoralis*	Temperature	97	2	48.66	0.001	0.999
	Location	97946	1	97945.86	2.968	0.093
	Temperature × Location	3682	2	1841.20	0.056	0.946
	Error	1254009	38	33000.22		
*D. aruanus*	Temperature	43917	2	21958.53	0.572	0.570
	Location	1639957	1	1639956.51	42.734	**<0.001**
	Temperature × Location	297730	2	148864.80	3.879	**0.031**
	Error	1189664	31	38376.26		
*P. moluccensis*	Temperature	321	2	160.55	0.012	0.988
	Location	1061906	1	1061906.26	76.833	**<0.001**
	Temperature × Location	65882	2	32940.98	2.383	0.103
	Error	677228	49	13820.98		
**(c) Aerobic scope**						
**Cardinalfish**						
*O. cyanosoma*	Temperature	407	2	203.35	0.013	0.987
	Location	1532	1	1531.98	0.100	0.754
	Temperature × Location	28012	2	14005.85	0.912	0.411
	Error	537256	35	15350.16		
*O. doederleini*	Temperature	2027	2	1013.29	0.070	0.932
	Location	201149	1	201149.11	13.966	**0.001**
	Temperature × Location	5977	2	2988.27	0.207	0.814
	Error	532891	37	14402.46		
**Damselfish**						
*A. polyacanthus*	Temperature	2803	2	1401.71	0.066	0.936
	Location	170277	1	170277.17	8.025	**0.007**
	Temperature × Location	39586	2	19793.03	0.933	0.402
	Error	806334	38	21219.33		
*C. atripectoralis*	Temperature	46	2	23.20	0.001	0.999
	Location	53358	1	53357.58	1.758	0.193
	Temperature × Location	5754	2	2877.19	0.095	0.910
	Error	1153314	38	30350.36		
*D. aruanus*	Temperature	23032	2	11515.79	0.361	0.700
	Location	1098903	1	1098902.59	34.451	**<0.001**
	Temperature × Location	141427	2	70713.44	2.217	0.126
	Error	988825	31	31897.60		
*P. moluccensis*	Temperature	190	2	94.94	0.005	0.995
	Location	521291	1	521290.56	29.347	**<0.001**
	Temperature × Location	34213	2	17106.60	0.963	0.389
	Error	870381	49	17762.88		

**Bold values** indicate significant test results.

### Cardinalfish

At common treatment temperatures (29, 31 and 32°C) the MO_2Rest_ values of one cardinalfish, *O. doederleini*, was approximately 20% higher for Heron Island fishes compared with Lizard Island fishes. Similarly the MO_2Max_ of this species was 20–30% greater in fish from Heron Island than in fish from Lizard Island (Fig. 2). Similar proportional differences among latitudes occurred for *O. cyanosoma*, but were not statistically significant (Table 2a–b). Heron Island fish were smaller than Lizard Island fish for this species (Table 1) affecting metabolic comparisons.

MO_2Rest_ of the cardinalfish species increased with temperature at both locations (Fig. 2, Table 3a). A 4°C increase in temperatures at Heron Island (from 27°C to 31°C) caused a 33% increase in MO_2Rest_ for *O. cyanosoma* and doubled the MO_2Rest_ in *O. doederleini*. A further temperature rise to 32°C increased MO_2Rest_ by an additional 25% in *O. cyanosoma* while MO_2Rest_ in *O. doederleini* was not significantly affected At Lizard Island a 4°C increase in temperature (from 29°C to 33°C) doubled MO_2Rest_ in both *O. cyanosoma* and *O. doederleini*.

**Table 3:** Multiple comparison test results comparing the effect of rising temperatures on (a) MO_2Rest_, (b) MO_2Max_ and (c) aerobic scope for two cardinalfish species and four damselfish species at two reef locations.

	Heron Island				Lizard Island			
	Control temperature = 27°C				Control temperature = 29°C			
*Species*	Treatment temp.	Mean difference	st. error	p	Treatment temp.	Mean difference	st. error	p
**(a) Resting metabolism**								
**Cardinalfish**								
*O. cyanosoma*	29	55.35	29.85	0.179	31	92.72	52.78	0.201
	31	100.32	28.91	**0.005**	32	210.57	52.78	**0.002**
	32	200.27	29.85	**<0.001**	33	159.75	57.82	**0.028**
*O. doederleini*	29	71.84	19.58	**0.003**	31	*Kruskal Wallis test:*		1.000
	31	133.48	19.58	**<0.001**	32	*H = 11.225, d.f. = 3,26.*		**0.039**
	32	163.84	20.93	**<0.001**	33	*p<0.05*		**0.035**
**Damselfish**								
*A. polyacanthus*	29	−7.22	21.01	0.975	31	158.50	39.62	**0.001**
	31	13.19	21.01	0.873	32	142.28	40.77	**0.004**
	32	50.63	20.13	0.051	33	173.56	40.77	**0.001**
*C. atripectoralis*	29	37.60	36.17	0.618	31	59.19	40.29	0.343
	31	106.84	37.74	**0.026**	32	55.63	41.45	0.413
	32	68.09	37.74	0.205	33	79.82	44.77	0.204
*D. aruanus*	29	69.09	51.56	0.423	31	108.50	47.53	0.077
	31	−15.27	51.56	0.981	32	66.35	47.53	0.379
	32	45.60	48.92	0.677	33	185.15	47.53	**0.002**
*P. moluccensis*	29	27.87	41.75	0.837	31	81.01	17.82	**<0.001**
	31	74.67	41.75	0.195	32	66.66	19.74	**0.005**
	32	102.30	44.29	0.071	33	125.00	19.74	**<0.001**
**(b) Maximum metabolism**								
**Cardinalfish**								
*O. cyanosoma*	29	157.38	57.08	**0.028**	31	−57.10	85.44	0.854
	31	254.91	55.27	**<0.001**	32	36.22	88.61	0.960
	32	247.89	57.08	**0.001**	33	−440.88	98.08	**<0.001**
*O. doederleini*	29	221.87	50.01	**0.000**	31	−82.19	78.89	0.600
	31	124.73	50.01	0.050	32	−7.25	86.42	1.000
	32	129.97	53.46	0.057	33	−165.13	90.64	0.192
**Damselfish**								
*A. polyacanthus*	29	262.67	44.36	**<0.001**	31	77.17	71.57	0.575
	31	315.81	44.36	**<0.001**	32	63.06	73.65	0.725
	32	243.98	42.51	**<0.001**	33	71.56	73.65	0.647
*C. atripectoralis*	29	335.28	108.88	**0.015**	31	−50.28	85.31	0.886
	31	430.80	113.62	**0.003**	32	31.62	87.78	0.970
	32	364.16	113.62	**0.011**	33	−178.28	94.82	0.171
*D. aruanus*	29	204.61	71.69	**0.034**	31	205.34	126.57	0.266
	31	108.88	71.69	0.330	32	73.72	126.57	0.888
	32	−46.98	68.01	0.828	33	80.14	126.57	0.862
*P. moluccensis*	29	261.80	63.07	**0.001**	31	153.68	42.54	**0.003**
	31	357.59	63.07	**<0.001**	32	−38.67	47.13	0.755
	32	288.36	66.90	**0.001**	33	170.63	47.13	**0.003**
**(c) Aerobic scope**								
**Cardinalfish**								
*O. cyanosoma*	29	102.02	55.44	0.183	31	−140.89	102.02	0.373
	31	154.58	53.68	**0.021**	32	−144.15	104.42	0.373
	32	47.61	55.44	0.724	33	−503.89	117.80	**0.001**
*O. doederleini*	29	150.03	52.13	**0.022**	31	−137.97	72.74	0.168
	31	−8.75	52.13	0.996	32	−144.96	79.69	0.193
	32	−33.88	55.73	0.868	33	−389.04	83.58	**<0.001**
**Damselfish**								
*A. polyacanthus*	29	269.88	43.17	**<0.001**	31	−81.33	81.02	0.625
	31	302.62	43.17	**<0.001**	32	−79.21	83.37	0.662
	32	193.35	41.37	**<0.001**	33	−102.00	83.37	0.479
*C. atripectoralis*	29	297.68	100.93	**0.020**	31	−109.46	84.95	0.445
	31	323.96	105.32	**0.015**	32	−24.00	87.42	0.986
	32	296.07	105.32	**0.027**	33	−258.10	94.42	**0.029**
*D. aruanus*	29	135.52	77.18	0.232	31	96.83	113.77	0.731
	31	124.14	77.18	0.290	32	7.37	113.77	1.000
	32	−92.58	73.22	0.466	33	−105.01	113.77	0.683
*P. moluccensis*	29	233.93	80.23	**0.018**	31	72.68	42.00	0.220
	31	282.93	80.23	**0.004**	32	−105.33	46.53	0.077
	32	186.06	85.10	0.091	33	45.63	46.53	0.648

For each location, treatment temperatures were tested against the control temperature using Dunnetts two-sided t test. Analyses were conducted upon the residuals of oxygen consumption regressed against test fish body mass for each location. Kruskal-Wallis test was used in the case of heterogeneous variances. **Bold values** indicate significant test results.

At both locations MO_2Max_ of cardinalfish were relatively stable within the temperature range of 29–32°C (Fig. 2). At Heron Island, both cardinalfish species had higher MO_2Max_ at 29°C than at 27°C, while no further increase occurred at higher temperatures (Fig. 2, Table 3b). In Lizard Island *O. cyanosoma*, a temperature increase to 33°C induced high mortality rates as MO_2Max_ fell to the MO_2Rest_ value (leading to a total loss of aerobic scope, see below). In Lizard Island *O. doederleini* maximum metabolic rates dropped by approximately 25%.

### Damselfish

While MO_2Max_ at common temperatures were higher in southern GBR populations for most of the damselfish, the effect of temperature on MO_2Rest_ was dependent upon location (Fig. 3). A significant interaction between location and temperature occurred in MO_2Rest_ for *D. aruanus* and *A. polyacanthus* (Table 2a). For *D. aruanus*, MO_2Rest_ at Lizard Island generally increased with temperature. However, MO_2Rest_ at Heron Island fluctuated as temperature increased (Fig. 3, Table 2a), leading to a significant interaction between location and temperature. MO_2Rest_ in *A. polyacanthus* populations rose with temperature but incremental increases were more variable in Heron Island fish than Lizard Island fish. Temperature and location did not interact for MO_2Rest_ of *P. moluccensis* and *C. atripectoralis*. Resting rates in *P. moluccensis* were higher in Heron Island populations than Lizard Island populations at all common temperatures (Fig. 3). In contrast, MO_2Rest_ values of *C. atripectoralis* were similar between locations at all common temperatures (Fig. 3).

**Figure 4:**
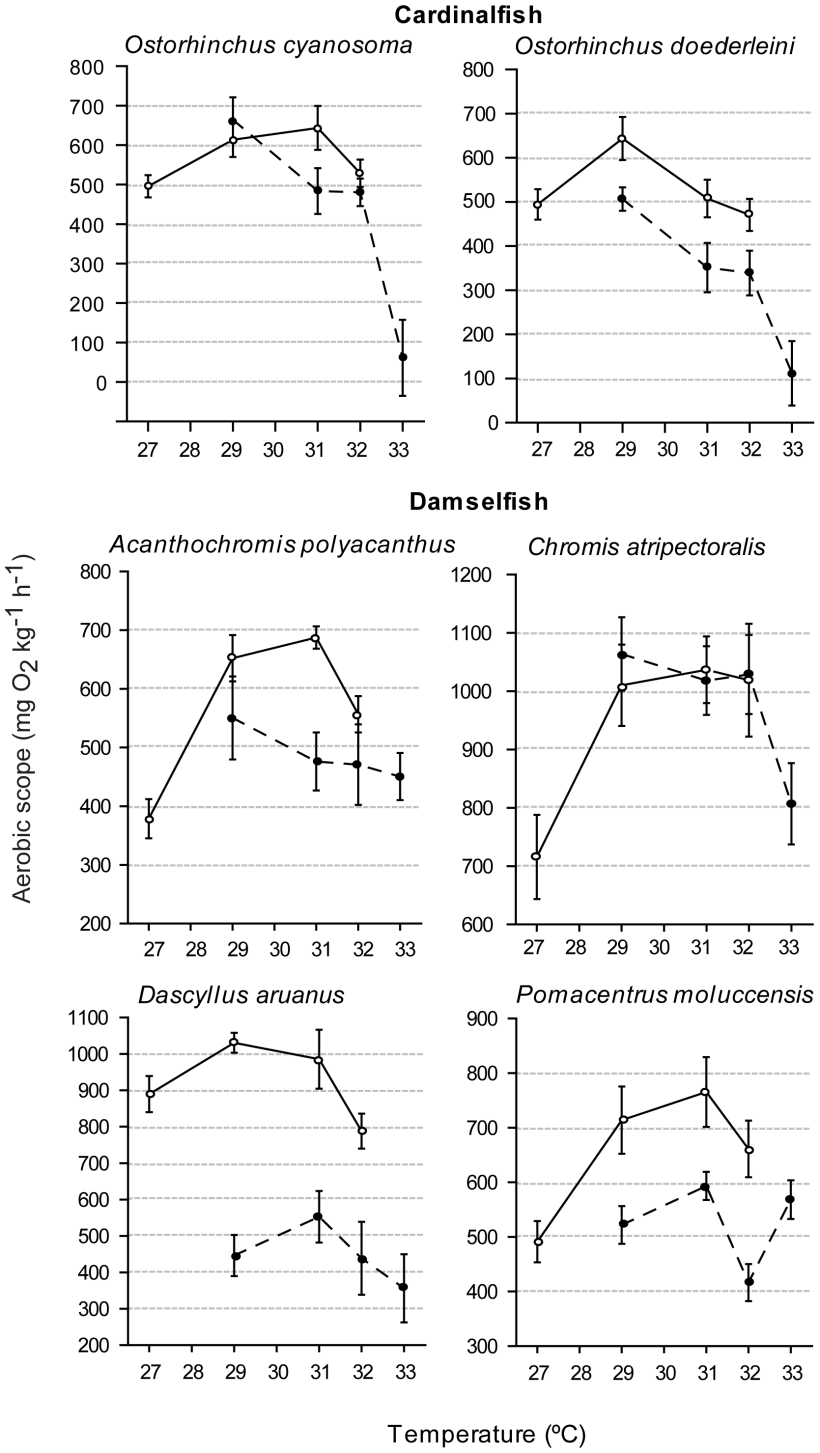
A latitudinal comparison of aerobic scope in cardinalfish and damselfish. Aerobic scope (MO_2Max_ - MO_2Rest_) in two cardinalfishes and four damselfishes from a high-latitude Great Barrier Reef location (Heron Island: open circles, solid lines) and a lower latitude Great Barrier Reef location (Lizard Island: solid circles, dashed lines). Values are means ± SE from 5–12 fish.

Increased temperatures of up to 4°C did not cause collapse of respiratory performance in damselfish at either location (Figs. 3–4). In general MO_2Max_ rates were stable at temperatures above the control average summer temperature. No significant decreases in MO_2Max_ were observed for damselfish (Table 3b). Southern populations of *D. aruanus* and northern populations of *C. atripectoralis* were the only ones to display any decline in MO_2Max_ when 29°C was exceeded (Fig. 3). Heron Island damselfish had lower MO_2Max_ at 27°C than at 29°C and in most cases MO_2Max_ was significantly lower at the control temperature (27°C) than at higher temperatures (Table 3b).

### Aerobic scope

The higher-latitude populations (Heron Island), had either higher or the same aerobic scope as lower-latitude populations (Lizard Island) (Fig. 4). The effects of location and temperature were statistically independent for all species (Table 2c). In total four species, one cardinalfish (*O. doederleini*) and three damselfish (*D. aruanus*, *P. moluccensis* and *A. polyacanthus*), displayed significantly higher aerobic scope at Heron Island than at Lizard Island (Table 2c, Fig. 4). The damselfish *D. aruanus* showed the greatest location difference, with aerobic scope 80–130% greater in Heron Island fish than Lizard Island fish, within the common temperature range of 29–32°C. Aerobic scope of *P. moluccensis* and *A. polyacanthus* was 30–60% and 18–45% times higher at Heron than Lizard Island, respectively. Aerobic scope of *O. doederleini* was 25–40% greater at Heron Island. No difference in aerobic scope between locations was observed for two species, the cardinalfish *O. cyanosoma* and the damselfish *C. atripectoralis* (Table 2c).

Temperature effects on aerobic scope within each location were variable among species and families (Table 3c, Fig. 4). A 4°C increase at Lizard Island caused a virtually complete loss of aerobic capacity in populations of the two cardinalfish species. Aerobic scope in southern populations initially rose with temperature before declining after thermal increases above 29–31°C. Compared to their 27°C control temperature, aerobic scope of *O. cyanosoma* was significantly higher at 31°C and *O. doederleini* was significantly higher at 29°C (Table 3c, Fig. 4). Indeed, no significant declines were seen in the aerobic scope of the Heron Island cardinalfish. However, like on Lizard Island, we did detect a high mortality of Heron Island cardinalfish kept at 33°C for a few days, precluding any experiments at this temperature.

With the exception of *D. aruanus*, the aerobic scopes of Heron Island damselfish were always higher at the experimentally elevated temperatures than at their average summer temperature control of 27°C (Fig. 4). Thus, Heron Island populations of *C. atripectoralis*, *P. moluccensis* and *A. polyacanthus* each had significantly higher aerobic scopes at 29, 31 and 32°C than at 27°C (Table 3c) *D. aruanus* was the only damselfish species at Heron Island for which elevating temperatures above the 27°C summer average did not significantly affect aerobic scope. For these fish, scope was 10% lower at 32°C than at the control temperature. Increased temperatures did not significantly affect the aerobic scope of Lizard Island damselfish except for *C. atripectoralis*, where a fall was seen at 33°C compared to 29°C (Table 3c).

In one cardinal fish (*O. doederleini*) and one damselfish (*P. moluccensis*), aerobic scope was virtually identical when measured at the control temperature of the respective location (Fig. 4). In other words, for these species 27°C values from Heron Island were the same as 29°C values from Lizard Island. This suggests that either local adaptation or thermal acclimation in these species leads to a constancy in aerobic scope at the prevailing summer temperature.

## Discussion

We detected significant differences in respiratory performance of reef fish populations at two widely separated locations on the Great Barrier Reef, but generally not as predicted by optimality models of thermal adaptation. Reef fish in the warmer, northern Great Barrier Reef location did not cope better with higher temperatures than their conspecifics in the cooler, southern region. Instead, the southern populations had either greater or equivalent aerobic scope than the northern populations when tested at common temperatures. This counter-gradient variation in absolute aerobic capacity was mostly driven by southern populations exhibiting up to 80% higher MO_2Max_ compared with the northern populations. Southern populations generally also had higher levels of MO_2Rest_ than northern populations when tested at common temperatures. The capacity to maintain aerobic scope as temperature increases is thought to be the primary mechanism determining the response of water breathing species to global warming [9], [34]. Due to their lower aerobic scope at higher temperatures, our results suggest that populations of tropical reef fishes living in already warm low-latitude locations will be more sensitive to future increases in ocean temperatures than conspecific populations living in cooler high-latitude locations.

Counter-gradient patterns in thermal performance typically involve some form of metabolic compensation for the negative effect of thermal gradients on particular traits [20], [21]. Such mechanisms may drive the higher maximal oxygen consumption observed in our higher latitude populations. There are at least four major physiological factors guiding the maximal rate of oxygen uptake in vertebrates [35]; (1) cardiac output that determines the rate of blood flow through the respiratory organ (e.g. gills) and perfusion of the rest of the body, (2) respiratory surface area (e.g. gill surface area), (3) the oxygen carrying capacity of the blood, which is dependent on hemoglobin concentration, and (4) the degree of downloading of oxygen from blood to the tissues. The higher absolute values for MO_2Max_ and aerobic scope displayed by the Heron Island populations in four out of six species could be explained by either larger respiratory surface areas or higher blood hemoglobin contents in these higher latitude populations. Fishes, like other vertebrates, can regulate blood hemoglobin content through erythropoesis [36] and recent studies have revealed that some fish have a significant capacity for changing the gill surface area both as adults [37] and during development [38], [39]. The higher MO_2Rest_ of Heron Island populations could also be explained by such differences, because a larger gill surface area will lead to higher energetic costs for maintaining ion-homeostasis [40] and a higher red blood cell content leads to increased blood viscosity and therefore higher energetic costs for maintaining blood circulation. The adaptive advantage for higher latitude populations having a higher MO_2Max_, a larger respiratory surface areas or a higher blood hemoglobin content is currently unknown, but could be related to detrimental effects of cooler water on one or more life-history traits. In particular, increased metabolic rate could help maintain growth and developmental rates in cooler water, as observed in some temperate water fishes and other aquatic species [21], [22]. Other factors, including differences in water flow regimes and short term temperature extremes, or differences in fish community structure (e.g. predators and competitors), might also contribute to differences in respiratory performance between fish from Lizard Island and Heron Island.

In contrast to the differences in aerobic scope seen in some Heron Island fish compared with Lizard Island fish, there was no clear evidence for differences in the thermal optima between fish from the two sites. However, further experiments would be required to confirm the apparent similarities in thermal optima between locations. Thermal optima of some ectotherms are fine-tuned to their local thermal environment. For example, the optimal thermal temperature of Pacific salmon populations varies in direct relationship with their historically experienced river temperatures [15]. If Heron Island fish populations were adapted to the average summer temperatures experienced at that location, MO_2Max_ and aerobic scope should have been highest at 27°C. Instead, aerobic scope for all six species at Heron Island was highest between 29–31°C, well above the average summer temperatures experienced at this latitude. Due to north - south dispersal gradients on the Great Barrier Reef (GBR) [27], Heron Island populations may receive considerable gene flow from northern populations causing their apparent thermal optimum to be more suited to the summer reef temperatures of lower latitude reefs (e.g. 29–31°C).

Two species did not exhibit a significant difference in aerobic scope between locations. One of these species, *O. cyanosoma*, exhibited similar responses in MO_2Rest_ and MO_2Max_ to the other species tested; however, the increase in MO_2Max_ at Heron Island compared with Lizard Island was primarily due to differences in body mass between locations. Heron Island *O. cyanosoma* were slightly smaller and thus had higher metabolic rates than Lizard Island fish. For *C. atripectoralis*, there was clearly no effect of location on MO_2Rest_, MO_2Max_, or aerobic scope. Within locations, temperature affected MO_2Rest_ and MO_2Max_ of *C. atripectoralis* as expected, but there was no difference in the magnitude of oxygen consumption between locations. Despite evidence of some genetic structure between *C. atripectoralis* populations on the northern and southern GBR [27], [28], our results reveal no apparent difference in the capacity for thermal acclimation in this species.

Overall, our results suggest that Heron Island fish may perform better under warmer water conditions likely to occur in the future. In contrast, increases in average summer temperatures of ≥2°C at Lizard Island are likely to affect community structure [26]. These lower-latitude populations appear to be living at or above their thermal optima and near their critical thermal limits. Whether the differences in respiratory performance we detected between locations are due to phenotypic or genotypic differences (ie. local adaptation, developmental plasticity, or a combination of the two) remains to be determined. Testing between these alternative will require sophisticated breeding experiments where offspring from the two populations are reared throughout their entire life-span at a range of different temperatures. There is some evidence that the differences in respiratory performance could have a least some genetic basis. Three of the damselfishes species tested here (*A. polyacanthus, C. atripectoralis, P. moluccensis*) exhibit genetic structure between the northern and southern GBR [27], [28], with the strongest structure exhibited by *A. polyacanthus*. Furthermore, some cardinalfishes, including *O. doederleini* [41] exhibit genetic structure similar to that exhibited by *A. polyacanthus* at local spatial scales. Thus, there is evidence for some level of genetic differentiation between Heron Island and Lizard Island populations, which is consistent with a hypothesis of local adaptation in respiratory performance. However, recent evidence for developmental plasticity in thermal acclimation by one of the species, *A. polyacanthus* (Donelson, unpublished data) suggests that there may also be flexibility in metabolic responses to temperature gradients that are established during the juvenile phase. Most likely, local genetic structure and developmental plasticity interact to generate the patterns of thermal performance we observed at each location.

It is evident that coral reef fish families and species differ in their tolerance to thermal changes. Greater aerobic sensitivity to increasing temperature was exhibited by the two cardinalfish species compared with the four damselfish species, at both Lizard Island and Heron Island. Familial differences may be related to behavioural differences in each group's activity patterns. Damselfish and cardinalfish are both well known as one of the most and one of the least active coral reef fish groups respectively [42]. Species with active behaviour, such as damselfish, are aerobically fitter and thereby predicted to cope better with increased metabolic energy demands (such as temperature) compared to groups, such as cardinalfish, with less daily energy expenditure [25], [43].

Geographic range differences among the study species may also contribute to differences in thermal tolerance. Lizard Island is close to the latitudinal extent of thermal and geographic ranges for the two cardinalfish species used in this study which are either not present or very rare in equatorial reef areas [44]. Other cardinalfish species whose distributions do extend to equatorial areas might have greater tolerance of higher temperatures. Amongst the damselfish, *P. moluccensis*, *A. polyacanthus* and *C. atripectoralis* have broader latitudinal ranges than *D. aruanus*. In the Great Barrier Reef region, the distribution of *D. aruanus* does not extend to the equator, being replaced by the sister species, *D. melanurus* in northern Papua New Guinea [45], [46]. In contrast, the other three damselfish species have distributions that extend to the equator. Despite these differences, we did not detect consistent differences in the respiratory performance of *D. aruanus* compared with the other damselfish species, except that it exhibited the largest change in aerobic scope between locations of any species. Clearly, determining if geographic ranges of tropical reef fishes are associated with respiratory thermal sensitivity will require more detailed knowledge on the geographical ranges and thermal performance curves of these species.

Whether warming oceans will shift the distributions of tropical marine species polewards depends strongly on the metabolic capacity of populations to keep pace with increased oxygen demand [9], [34]. If the strong counter-gradient patterns seen here are prevalent among reef fish, and other tropical marine ectotherms, then lower latitude populations inhabiting warmer waters will be more sensitive to global warming than populations of the same species at higher latitudes. Where dispersal mechanisms permit we might expect species with distributions currently centered in low latitudes to expand their distribution polewards. The long term viability of populations at low latitudes will depend on their ability to acclimate or adapt to warmer seas. Given the lack of evidence we detected for fine-tuning of thermal optima to match average or maximum summer temperatures, prospects for rapid adaptation to warming ocean temperatures by low-latitude populations may be limited.
